# Determinants of household dropout from community-based health insurance program in northwest Ethiopia; A community-based case-control study

**DOI:** 10.1371/journal.pone.0276676

**Published:** 2023-01-11

**Authors:** Gizachew Tadesse Wassie, Getasew Tadesse, Gebeyehu Tsega Nebeb, Amare Alemu Melese, Agumas Fentahun Ayalew, Getasew Mulat Bantie

**Affiliations:** 1 Department of Epidemiology, College of Medicine and Health Science, Bahir Dar University, Bahir Dar, Ethiopia; 2 Department of Health Economics, management and Policy, College of Medicine and Health Science, Bahir Dar University, Bahir Dar, Ethiopia; 3 AAM: Food Safety, and Microbiology Reference Laboratory, Ethiopian Public Health Institute, Addis Ababa, Ethiopia; 4 AFA: Department of Epidemiology, College of Health Science, Injibara University, Injibara, Ethiopia; 5 GMB: Amhara National Regional State Public Health Institute, Bahir Dar City, Ethiopia; 6 GMB: Department of Public Health, Faculty of Community Health, Alkan Health Science Business and Technology College, Bahir Dar city, Ethiopia; Purdue University, UNITED STATES

## Abstract

**Background:**

Community-Based Health Insurance (CBHI) is an evolving program for delivering financial protection against the cost of illness and enhancing access to quality health services for low-income informal households.

**Objective:**

The study aimed to identify determinants of household dropout from a CBHI program in Mecha district, North West Ethiopia, 2019.

**Methods:**

A community-based case-control study was conducted in the Mecha district from March 10 to June 10, 2018. The final sample was 634 (317 cases and 317 controls) determined by the two-population proportion formula, and these samples were selected using a multi-stage sampling technique. The collected data was entered into Epi-data version 3.1 and analyzed using R version 4.0 software. Descriptive statistics computed. A simple logistic analysis was run (at 95% CI and p-value < 0.05) to identify the determinants for the dropout from CBHI.

**Results:**

Poor perceived quality of care (AOR = 3.66; 95%CI: 2.35, 5.69), low knowledge of community-based health insurance (AOR = 6.02; 95%CI: 2.97, 12.26), no active community communication (AOR = 5.41; 95%CI: 3.29, 8.90) no chronic illness (AOR = 10.82; 95%CI: 5.52, 21.21) premium fee is not affordable (AOR = 2.35; 95%CI: 1.47, 3.77), and out of pocket money not reimbursed (AOR = 9.37; 95%CI: 4.44, 19.77) were the determinants for the dropout from CBHI.

**Conclusions:**

Poor perceived quality of care, low knowledge of CBHI, no active community participation, no chronic illness, premium fees are not affordable, and out-of-pocket money not reimbursed were the determinants for the dropout from CBHI.

## Background

Community-Based Health Insurance (CBHI) is a not-for-profit and an emerging concept for providing financial protection against the cost of illness and enhancing access to quality health services for low-income households excluded from formal insurance [1, 2]. The members of CBHI schemes play essential roles in risk-sharing, mobilizing, pooling, allocating, managing, and supervising healthcare resources [3, 4].

According to the World Health Organization, everyone should access high-quality, consumer-friendly healthcare services regardless of financial situation [5]. However, access to high-quality, consumer-friendly healthcare services is limited in Sub-Saharan Africa [6–8].

Ethiopia has a low living standard, a high morbidity and sickness rate, and a lack of health care financing [9]. To address these issues, Ethiopia designed community-based health insurance in June 2011 as a pilot in 13 districts found in four regions, namely, Tigray, Amahara, Oromiya, and South Nations and Nationalities people’s Region (SNNPR) [10], and been striving to expand it across the country [11]. Unlike the social health insurance scheme, joining the CBHI is based on the voluntary decision of the households. In the pilot districts, families that enter the community-based health insurance are expected to pay 180 Ethiopian Birr (10.4 US dollars) annually as a premium. However, the members’ contribution varies among the pilot districts, ranging from 34.4 ETB to 132 ETB [12].

Similarly, the Ethiopian government has launched a national health sector transformation plan for 2016–2020. It is working harder than ever by increasing healthcare financing, quality, equity, human resources, and strategies that enable universal health coverage [11].

The community-based health insurance scheme provides many benefits of health care service utilization at public health facilities [11]. However, the Scheme excludes some health care packages like treatment abroad, kidney dialysis, artificial teeth treatment, and plastic surgery [11]. In 2015, the Ethiopian government expanded community-based health insurance to 320 districts [13]. Though the initial uptake of CBHI was encouraging in the Mecha district, the dropout rate from CBHI membership is increasing [14].

Different indigenous studies reported that older age, female sex, educational status, household family size, poor perceived quality of health care, low income, received promised Benefit package, providers attitude, trust in the health facility, trust in the Scheme, knowledge of health insurance were the factors associated with CBHI dropouts, length of enrollment, households visit the health facilities, have no access to the hospital, and official position holder for decision–making in the homes were associated with CBHI dropouts [15–17].

The motive for conducting this study was to assist in the sustainment of the CBHI scheme by identifying the determinants for the CBHI dropouts. Thus, identifying the factors contributing to CBHI dropout may improve the program’s sustainability. It is critical for policymakers at the national and regional levels to develop evidence-based CBHI intervention strategies that focus on and address the determinants of CBHI dropouts.

## Methods

A community-based case-control study was conducted among Mecha District residents from March 10 to June 10, 2018. It is 530 kilometres northwest of Ethiopia’s capital, Addis Ababa and is divided into 43 kebeles (The minor administrative units) for administrative purposes. The district has a population of 290,080 and 66,107 households [18]. In 2017, 34,170 households were members of the CBHI scheme, of which 11,466 did not renew their membership, a dropout rate of 34%. This district has ten health centers and one primary hospital [19].

### Population

All households enrolled in the CBHI scheme in Mecha District were the source population. The families enrolled in the twelve selected kebeles of the Mecha District were included in the study.

### Sample size determination

The sample size was determined using Epi- Info version 7 software, with the stat calc Fleiss W/CC model. The identified determinants for CBHI scheme dropout (reviewed from a previously published article) were illness experience in the household, age, and trustworthiness of scheme management [20]. Then, considering these determinants’ results, the predictor with the largest estimated sample size was chosen. Common assumptions include a 1:1 case-control ratio, 80% power, adjusted odds ratio at 95% confidence interval, 5% precision, a design effect of 2, and a 10% non-response rate. Finally, 634 (317-cases and 317-controls) households were included in the study (Table 1).

**Table 1:** Determinants for the sample size estimation of CBHI dropouts in Mecha District, northwest Ethiopia, 2018.

Exposure	Proportion (%) controls exposed	AOR (95% CI)	Power	Required Samples		Total samples	Design effect (2)	Non-response rate (10%)	Final Sample Size	Reference
				Cases	Controls					
Illness experience in household	39.6	2.00	80%	144	144	288	576	57.6	634	Mladovsky P, 2014
Age of HH (highest) category	27.88	0.31	80%	93	93	186	372	37.2	410	
Trustworthiness of scheme management	69.21	4.00	80%	66	66	132	264	26.4	291	

### Sampling procedure

The study participants were drawn using a multi-stage sampling technique. In the first stage, 12 kebeles were selected randomly (lottery method) out of 43 kebeles. The second stage involved the selection of households from among the twelve kebeles. The list of households for cases and controls was obtained from each kebele’s administration household record list, which was used as a sampling frame. The sample size was proportionally allocated to selected kebeles based on each kebele’s number of households. The cases and controls were then randomly selected from each Keble’s CBHI registration book using a simple random sampling method.

#### Variables of the study

*Dependent variable*. Dropout of CBHI membership.

*Independent variables*. **Socio-demographic characteristics**: Sex of head, education status of the head, age of the head, marital status of the household head, the average annual income of the household, and family size.

**Household health status and healthcare utilization**: Health facility visits, illness experience in the household, presence of chronic illness in the household, and inpatient care use.

**Physical access to health care**: Travel time to the health center and public hospital.

**Perceived quality of care linked to CBHI**: health worker impartiality, professional ethics, competency, availability of ordered diagnostic equipment, availability of prescribed basic drugs, availability of all benefit packages, waiting time to get a consultation, and diagnostic results.

*Knowledge of CBHI-related characteristics*. **CBHI Scheme-related characteristics**: premium fee affordability, community participation, CBHI management, staff trustworthiness, renewal time appropriateness, Scheme’s referral procedure, and reimbursement.

*Operational definitions*. **Cases**: households that previously participated in the CBHI scheme but have not paid their annual CBHI renewal contribution fees and hence do not have access to the benefits of the CBHI packages.

**Controls**: Households that had paid their annual CBHI renewal contribution fee and had access to the benefits of CBHI packages.

**Perceived quality of care**: Those household heads that scored 50^th^ percentile or more on any of the eight perceived quality of care assessment questions were considered to have a good perceived quality of care. However, household heads that scored below the 50^th^ percentile were considered to have a poor perceived quality of care.

**Knowledge of CBHI**: The respondents were considered to have high knowledge of CBHI when they answered all five knowledge-assessing questions correctly. The respondents were also considered to have medium knowledge of CBHI when they correctly answered three or four of the knowledge-assessing questions. Otherwise, they were considered to have low knowledge.

**Wealth index**: Consumer items and household assets were used to calculate the wealth index. The households were then graded from lowest to greatest score and divided into quintiles, representing 20% of the population [21].

**Kebele**: The smallest administrative unit in Ethiopia [22].

### Data collection tool and procedure

The data was collected via primary and secondary data sources. The secondary sources of data were retrieved from the CBHI registration book. Then, the primary data was collected using a pretested structured questionnaire adapted from a variety of literature, ’S1 Files’. The questionnaire comprises socio-demographics, physical access to health institutions, perceived quality of care, health status and utilization, knowledge of CBHI, and scheme-related characteristics. It was translated into Amharic (the indigenous) language by an independent translator (Ph.D. in linguistics). Then, back to English to check for consistency. The principal investigator gave two days of rigorous training to six enumerators (BSc in clinical nursing) and two supervisors (MPH in Epidemiology). The enumerators conducted role-play before the actual data collection period. The enumerators and the project investigators performed pretest activities in five percent of the total sample size before the data collection. Then, ambiguous sentences were explicitly rephrased. The internal consistency (Cronbach alpha) level of the pretest of stunting was 0.87. Finally, the data were collected using the Amharic version of the questionnaire. The supervisor and principal investigator examined each questionnaire daily for completeness and consistency. Then, appropriate feedback was given to the enumerators.

### Data management and analysis

Data entry, cleaning, and coding were performed using Epi-data version 3.1 software, and analysis was done using R version 4.0 software. Descriptive statistics were computed and presented using tables and texts. A p-value of less than 0.2 in the bivariate analysis was considered for variables to be included in the multivariable logistic regression analysis. Variables with a p-value of less than 0.05 on multivariate analysis were considered statistically significant predictors of dropout from the CBHI scheme.

### Ethical considerations

The Institutional Review Board of Bahir Dar University gave an ethical approval with Protocol number: 0158/18-09. Prior to the data collection period, the Amhara National Regional State Health Bureau and the Public Health institute both provided consent letters. The zonal health department and the Mecha district health and CBHI offices also filed a letter of support. Before any data was gathered, the respondents were told of the study’s purpose and were given written informed consent in Amharic. Those who had fallen out of community-based health insurance were strongly advised to re-enroll.

## Results

### Socio-demographic characteristics of the participants

A total of 634 (317 cases and 317 controls) study households were interviewed and yielded a response rate of 100%. Most cases (84.9%) and controls (87.7%) were male household heads. A more significant portion of cases (45.7%) and controls (45.4%) were in the age group of 30–39 years. More than half of the cases and controls could not read and write. Approximately 88% of the cases and controls were married. More than 88% of the cases and controls were farmers, and about 65% of the cases and 59% of the controls had a family member of six or more (Table 2).

**Table 2:** Socio-demographic characteristics of the household heads in Mecha District, 2018.

Variables	Categories	CBHI dropout			X^2^, P-Value
		Cases N (%)	Controls N (%)	Total % N = 634	
Sex	Male	269 (84.9)	278 (87.7)	547 (86.3)	1.079, 0.299
	Female	48 (15.1)	39 (12.3)	87 (13.7)	
Age (in years)	20–29	58 (18.3)	68 (21.5)	126 (20.0)	3.73, 0.29
	30–39	145 (45.7)	144 (45.4)	289 (45.6)	
	40–49	107 (33.8)	92 (29.0)	199 (31.4)	
	50–59	7 (2.2)	13 (4.1)	20 (3.1)	
Marital status	Single	0	4 (1.3)	4 (0.6)	
	Married	280 (88.3)	279 (88.0)	559 (88.2)	
	Divorced	21 (6.6)	20 (6.3)	41 (6.5)	
	Windowed	16 (5.0)	14 (4.4)	30 (4.7)	
Educational status	Can’t read and write	167 (52.7)	178 (56.1)	345 (54.4)	11.7, 0.003
	Can read and write*	110 (34.7)	76 (24.0)	186 (29.3)	
	Primary and above	40 (12.6)	63 (19.9)	103 (16.3)	
Family size	≤ 5	113 (35.6)	131 (41.3)	244 (38.5)	2.16, 0.14
	>5	204 (64.4)	186 (58.7)	390 (61.5)	
Occupation	Farmer	287 (90.5)	279 (88.0)	566 (89.3)	1.05, 0.305
	Merchant	30 (9.5)	38 (12.0)	68 (10.7)	
Annual household income	Quartile 1	74 (23.3)	85 (26.8)	159 (25.1)	8.303, 0.04
	Quartile 2	74 (23.3)	98 (30.9)	172 (27.1)	
	Quartile 3	87 (27.4)	72 (22.7)	159 (25.1)	
	Quartile 4	82 (25.9)	62 (19.6)	144 (22.7)	

Can read and write*: This implies that the respondents can read and write but didn’t attend formal education.

## Physical access to health institutions and perceived quality of care

Accordingly, about 46% of the cases and 42% of the controls take 30–60 minutes to arrive at the nearby health center. However, 60% of the cases and 50% of controls spend 1–2 hours reaching the nearby hospital. About 67% of the cases and 61% of the controls responded that the health facilities were far from their houses. And 84% of the cases and 75% of the controls were also waiting a long time to get health care. About 68% of cases and 44% of controls didn’t get the prescribed drugs in the health facility. About 84% of the cases and 49% of the control perceived the contracted health facilities to give quality health care (Table 3).

**Table 3:** Physical accessibility and perceived quality of care in the Mecha district in 2018.

Variables	Categories	CBHI dropout			X^2^, P-Value
		Cases N (%)	Controls N (%)	Total N (%)	
Travel time to reach the nearest health center	<30 minutes	31 (9.8)	42 (13.2)	73 (11.5)	7.730, 0.052
	30–60 minutes	146 (46.1)	133 (42.0)	279 (44.0)	
	1–2 hours	138 (43.5)	132 (41.6)	270 (42.6)	
	>2 hours	2 (0.6)	10 (3.2)	12 (1.9)	
Travel time to reach the nearest hospital	<30 minutes	7 (2.2)	20 (6.4)	27 (4.3)	15.630, 0.001
	30–60 minutes	102 (32.2)	104 (32.8)	206 (32.5)	
	1–2 hours	191 (60.3)	158 (49.8)	349 (55.0)	
	>2 hours	17 (5.4)	35 (11.0)	52 (8.2)	
Impartiality	Yes	164 (51.7)	222 (70)	386 (60.9)	22.28, 0.0001
	No	153 (48.3)	95 (30)	248 (39.1)	
The health care workers are ethical	Yes	156 (49.2)	214 (67.5)	370 (58.4)	21.38, 0.0001
	No	161 (50.8)	103 (32.5)	264 (41.6)	
The health professionals are competent	Yes	178 (56.2)	232 (73.2)	410 (64.7)	20.13, 0.0001
	No	139 (43.8)	85 (26.8)	224 (35.3)	
The health facilities have quality laboratory services	Yes	138 (43.5)	195 (61.5)	333 (52.5)	20.55, 0.0001
	No	179 (56.5)	122 (38.5)	301 (47.5)	
The health facilities have enough drugs	Yes	103 (32.5)	177 (55.8)	280 (44.2)	35.03, 0.0001
	No	214 (67.5)	140 (44.2)	354 (55.8)	
The health facilities are far from their house	Yes	211 (66.6)	193 (60.9)	404 (63.7)	2.211, 0.137
	No	106 (33.4)	124 (39.1)	230 (36.3)	
You had to wait a long time to get health care	Yes	267 (84.2)	237 (74.8)	504 (79.5)	8.709, 0.003
	No	50 (15.8)	80 (25.2)	130 (20.5)	
The members of the CBHI scheme got all the benefit packages from the health facilities	Yes	87 (27.4)	149 (47.0)	236 (37.2)	0.406, 0.524
	No	230 (72.6)	168 (53.0)	398 (62.8)	
The CBHI members’ perception of quality care	Poor	265 (83.6)	156 (49.2)	421 (66.4)	84.00, 0.0001
	Good	52 (16.4)	161 (50.8)	213 (33.6)	

### Characteristics associated with health status and health care utilization

Illness happened in 89% of the cases and 86% of control households for the last year. Eighteen percent of the cases and 28% of the controls had chronic illnesses in their household. Hypertension was the predominant chronic disease among cases (47.4%) and controls (46.6%). More than 90% of the cases and controls had ever visited government health institutions in the last year. Of these, 24% of the cases and 28% of the controls got inpatient care within a year (Table 4).

**Table 4:** Health care utilization-related characteristics of the Mecha district, 2018.

Variables	CBHI dropout		TotalN (%)	X^2^, P-Value
	Cases N (%)	Controls N (%)		
**Illness experience in household for the last one year**				
Yes	282 (89.0)	273 (86.1)	555 (87.5)	1.171, 0.279
No	35 (11.0)	44 (13.9)	79 (12.5)	
**Who got sick**				
Children	121 (38.2)	83 (26.2)	204 (32.2)	16.641, 0.001
Adults	155 (48.9)	160 (50.5)	315 (49.7)	
Elderly (> 65 years)	2 (0.6)	4 (1.3)	6 (0.9)	
Other family members	39 (12.3)	70 (22.1)	109 (17.2)	
**Chronic illness**				
Yes	57 (18)	88 (27.8)	145 (22.9)	8.593, 0.003
No	260 (82)	229 (72.2)	489 (77.1)	
**Types of chronic illness**				
Hypertension	27 (47.4)	41 (46.6)	68 (46.9)	10.772, 0.013
Diabetes Mellitus	12 (21.1)	5 (5.7)	17 (11.7)	
Respiratory diseases	0 (0)	4 (4.5)	4 (2.8)	
Others	18 (31.5)	38.(43.2)	56 (38.6)	
**Have you ever visited health institutions in the last year**				
Yes	288 (90.9)	291 (91.8)	579 (91.3)	0.179, 0.672
No	29 (9.1)	26 (8.2)	55 (8.7)	
**Inpatient care in the last 12 months**				
Yes	69 (24.0)	82 (28.2)	151 (26.1)	1.337, 0.248
No	219 (76.0)	209 (71.8)	428 (73.9)	

### Knowledge of CBHI and the scheme related characteristics

Nearly 77% of the cases and 84% of the controls perceived that they had enough information about CBHI. About 85% of the cases and 76% of the controls got information from kebele mobilizers. Nearly 34% of the cases and 14% of the controls have low knowledge of CBHI. Similarly, 40% of cases and 19% of the controls thought the CBHI contribution fee was not affordable. 25% of the cases and 12% of the controls spent their out-of-pocket money, and the CBHI scheme didn’t reimburse them. The reason given to them for non-reimbursement by officials was unfulfilled documentation (75%), followed by a failure to follow the referral system (25%) (Table 5).

**Table 5:** Knowledge of CBHI related characteristics in Mecha district, 2018.

Variables	CBHI		TotalN (%)	X^2^, P-Value
	Cases N (%)	Controls N (%)		
**Did you believe you had enough information to apply for a CBHI**				
Yes	245 (77.3)	266 (83.9)	511 (80.6)	4.448, 0.035
No	72 (22.7)	51 (16.1)	123 (19.4)	
**Source of information**				
kebele mobilizer	208 (84.9)	201 (75.6)	409 (80.4)	4.084, 0.130
Health extension workers	131 (53.5)	116 (43.6)	247 (49.0)	
Program workers	42 (17.1)	64 (24.1)	106 (21.0)	
District leaders	2 (0.9)	18 (6.8)	20 (3.9)	
Mass Media	27 (11)	40 (15)	67 (13.2)	
**Community participation**				
Yes	92 (29)	191 (60.3)	283 (44.6)	62.56, 0.0001
No	225 (71)	126 (39.7)	351 (55.4)	
**Who covers health service costs for members**				
The CBHI scheme	176 (55.5)	200 (63.1)	376 (59.3)	3.774, 0.152
Patient	108 (34.1)	89 (28.1)	197 (31.1)	
Health service providers	33 (10.4)	28 (8.8)	61 (9.6)	
**Only sick people join the CBHI program**				
Yes	17 (5.4)	45 (14.2)	62 (9.8)	14.02, 0.0001
No	300 (94.6)	272 (85.8)	572 (90.2)	
**Can premium have returned**				
Yes	35 (11)	32 (10.1)	67 (10.6)	0.150, 0.698
No	282 (89)	285 (89.9)	567 (89.4)	
**Participants’ knowledge of CBHI**				
Low	107 (33.8)	43 (13.6)	150 (23.7)	36.78, 0.0001
Medium	127 (40.1)	178 (56.2)	305 (48.1)	
High	83 (26.2)	96 (30.3)	179 (28.2)	
**Do you think the contribution fee is affordable**				
Yes	190 (59.9)	256 (80.8)	446 (70.3)	32.94, 0.0001
No	127 (40.1)	61 (19.2)	188 (29.7)	
**Do you trust scheme management**				
Yes	305 (96.2)	299 (94.3)	604 (95.3)	1.260, 0.262
No	12 (3.8)	18 (5.7)	30 (4.7)	
**Do you think renewal time is appropriate**				
Yes	307 (96.8)	299 (94.3)	606 (95.6)	2.391, 0.122
No	10 (3.2)	18 (5.7)	28 (4.4)	
**Do you have not reimbursed out-of-pocket money**				
Yes	78 (24.6)	38 (12.0)	116 (18.3)	16.88, 0.001
No	239 (75.4)	279 (88.0)	518 (81.7)	
**The reasons for the non-reimbursement of out-of-pocket money**				
Unfulfilled documentation	58 (74.4)	29 (76.3)	87 (75)	0.052, 0.819
Didn’t follow referral system	20 (25.6)	9 (23.7)	29 (25)	
**Do you believe the referral system is suitable**				
Yes	211 (66.6)	249 (78.5)	460 (72.6)	11.43, 0.001
No	106 (33.4)	68 (21.5)	174 (27.4)	
**The reasons for the non-suitability of the referral system**				
Delay to refer	84 (79.2)	51 (75.0)	135 (77.6)	0.429, 0.512
It restricts our preference	22 (20.8)	17 (25.0)	39 (22.4)	

### Determinants of dropout from CBHI in Mecha District

Those households having a poor perception of health care quality in the contracted health facilities were believed to be dropping out of the CBHI scheme. However, age changes probable confounder.

Poor perceived quality of health care is a significant risk factor for CBHI dropouts in all age-specific stratum though the intensity is highest in the lowest and highest age categories of 20–29 years [OR = 16.96 (95%CI: 6.34, 45.3)], 30–39 years [OR = 5.26 (95%CI: 3.08, 8.99), 40–49 years [OR = 2.24 (95%CI: 1.16, 4.34)], 50–59 years [OR = 13.50 (95%CI: 1.19, 152.21)] (Table 6); COR = 5.26 (95%CI: 3.63, 7.61) (Table 7). Hence, age is the effect modifier for this study but not a confounder factor as the stratum specific and the crude OR different.

**Table 6:** Crude and stratified analysis to quantify the associations between the exposure variables and the probable confounders (age, sex, and income) among 634 CBHI enrolled households in Mecha district, Northwest Ethiopia.

**20–29 years**						**30–39 Years**				**40–49 Years**				**50–59 Years**			
Stratified analysis by age		Case n (%)	Control n (%)	Odds Ratio	95% CI	Case n (%)	Control n (%)	Odds Ratio	95% CI	Case n (%)	Control n (%)	Odds Ratio	95% CI	Case n (%)	Control n (%)	Odds Ratio	95% CI
Perceived quality of care	Poor	52 (69.3)	23	16.96	6.34, 45.3	119 (64)	67	5.26	3.08, 8.99	88 (58.7)	62	2.24	1.16, 4.34	6 (60)	4	13.50	1.19, 152.2
	Good ^R^	6 (11.8)	45			26 (25.2)	77			19 (38.8)	30			1(10)	9		
Knowledge of CBHI	Low	20 (80)	5	5.50	1.21, 36.62	36 (67.9)	17	2.56	0.28, 1.72	47 (72.3)	18	1.92	1.08, 3.42	4 (57.1)	3	2.52	2.00, 10.1
	Medium	14 (31.8)	30	0.64	0.44, 0.93	71 (46.7)	81	1.06	0.46, 1.53	41 (40.6)	60	0.50	0.29, 0.87	1 (12.5)	7	0.21	0.01, 4.23
	High ^R^	24 (42.1)	33			38 (45.2)	46			19 (57.6)	14			2 (40)	3		
Active community participation	Yes ^R^	19 (28.8)	47	4.59	2.17, 9.75	41 (30.4)	94	4.77	2.89, 7.85	30 (41.7)	42	2.16	1.19, 3.88	2 (20)	8	4.00	0.55, 29.1
	No	39 (65)	21			104 (67.5)	50			77 (60.6)	50			5 (50)	5		
Chronic Illness	Yes ^R^	10 (43.5)	13	1.14	0.46, 2.82	21 (37.5)	35	1.89	1.04, 3.45	26 (41.3)	37	2.09	1.14, 3.85	0 (0)	3	----	----
	No	48 (46.6)	55			124 (53.2)	109			81 (59.6)	55			7 (41.2)	10		
Premium fee affordability	Yes ^R^	7 (70)	3	0.34	0.08, 1.37	14 (53.8)	12	0.85	0.38, 1.91	14 (48.3)	15	1.29	0.59, 2.85	0 (0)	2	----	----
	No	51 (44)	65			131 (49.8)	132			93 (54.7)	77			7 (38.9)	11		
Non-reimbursed OOPM	Yes	15 (71.4)	6	3.61	1.29, 10.1	32 (69.6)	14	2.63	1.34, 5.12	30 (65.2)	16	1.85	0.93, 3.67	1 (33.3)	2	0.92	0.07, 12.33
	No ^R^	43 (41)	62			113 (46.5)	130			77 (50.3)	76			6 (35.3)	11		
**Quartile 1**						**Quartile 2**				**Quartile 3**				**Quartile 4**			
Stratified by Income		Case n (%)	Control n (%)	Odds Ratio	95% CI	Case n (%)	Control n (%)	Odds Ratio	95% CI	Case n (%)	Control n (%)	Odds Ratio	95% CI	Case n (%)	Control n (%)	Odds Ratio	95% CI
Perceived quality of care	Poor	65 (51.2)	62	2.68	1.15, 6.24	74 (91.4)	7	----	----	87 (56.1)	68	----	----	39 (67.2)	19	2.05	1.03, 4.10
	Good ^R^	9 (28.1)	23			0 (0)	91			0 (0)	4			43 (50)	43		
Knowledge of CBHI	Low	34 (77.3)	10	2.83	0.91, 8.87	1 (100)	0	----	----	31 (50)	31	0.42	0.19, 0.91	41 (95.3)	2	76.2	0.06, 95451
	Medium	28 (30.1)	65	0.36	0.14, 0.95	33 (40.7)	48	0.86	0.78, 0.94	32 (50.8)	31	0.43	0.2, 0.91	34 (50)	34	3.71	1.03, 13.1
	High ^R^	12 (54.5)	10			40 (44.4)	50			24 (70.6)	10			7 (21.2)	26		
Active community participation	Yes ^R^	0 (0)	35	----	----	42 (30)	98	----	----	40 (97.6)	1	0.02	0.002, 0.125	10 (14.9)	57	82.08	26.56, 253.67
	No	74 (59.7)	50			32 (100)	0			47 (39.8)	71			72 (93.5)	5		
Chronic Illness	Yes ^R^	54 (69.2)	24	0.15	0.07, 0.29	0 (0)	0	----	----	3 (4.9)	58	116.0	31.89, 421.9	0 (0)	6	----	----
	No	20 (24.7)	61			74 (43)	98			84 (85.7)	14			82 (59.4)	56		
Premium fee affordability	Yes ^R^	57 (52.3)	52	0.47	0.23, 0.94	7 (6.7)	98	----	----	74 (61.2)	47	0.33	0.15, 0.71	52 (46.8)	59	11.35	3.27, 39.36
	No	17 (34)	33			67 (100)	0			13 (34.2)	25			30 (90.9)	3		
Non-reimbursed OOPM	Yes	33 (68.8)	15	3.76	1.83, 7.73	43 (87.8)	6	21.3	8.26, 54.8	1 (5.6)	17	0.04	0.005, 0.3	1 (100)	0	----	----
	No ^R^	41 (36.9)	70			31 (25.2)	92			86 (61)	55			81 (56.6)	62		

**Male**						**Female**			
Stratified by sex		Case n (%)	Control n (%)	OR	95% CI	Case n (%)	Control n (%)	OR	95% CI
Perceived quality of care	Poor	217 (59.8)	146	3.773	2.57, 5.54	48 (82.8)	10	----	----
	Good ^R^	52 (28.3)	132			0 (0)	29		
Knowledge of CBHI	Low	104 (71.2)	42	3.38	1.88, 6.09	3 (75)	1	1.50	0.57, 3.95
	Medium	102 (40.5)	150	0.93	0.90, 0.97	25 (47.2)	28	0.45	0.31, 0.65
	High ^R^	63 (42.3)	86			20 (66.7)	10		
Active community participation	Yes ^R^	61 (27.9)	158	4.49	3.09, 6.51	31 (48.4)	33	3.02	1.05, 8.64
	No	208 (63.4)	120			17 (73.9)	6		
Chronic illness	Yes ^R^	57 (40.7)	83	1.58	1.07, 2.34	0 (0)	5	----	----
	No	212 (52.1)	195			48 (58.5)	34		
Premium fee affordable	Yes ^R^	174 (44.1)	221	2.12	1.44, 3.11	16 (314)	35	17.5	5.29, 57.87
	No	95 (62.5)	57			32 (88.9)	4		
Non-reimbursed out-of-pocket money	Yes	53 (59.6)	36	1.65	1.04, 2.62	25 (92.6)	2	20.11	4.35, 92.99
	No ^R^	216 (47.2)	242			23 (38.3)	37		

COR: crude odds ratio; OOPM: Out-of-pocket-money

**Table 7:** Adjusted odds ratios estimated via binary logistic regression to quantify the associations between the exposure variables and the confounder variables (age, sex, and income) among 634 CBHI enrolled households in Mecha district, Northwest Ethiopia.

Variable	Category	CBHI Dropout		COR (95%CI)	AOR (95%CI)	P-value
		Case	Control			
Educational status	Unable to read and write	167	178	1.00	1.00	
	Able to read and write	109	76	**1.54 (1.08, 2.21)**	0.85 (0.53, 1.37)	0.514
	Primary and above	41	63	0.67 (0.43, 1.06)	0.89 (0.49, 1.59)	0.695
Time spent to arrive at the nearby hospital	< 30 minutes	7	20	1.00	1.00	
	30–60 minutes	102	104	**2.81 (1.14, 6.91)**	1.75 (0.56, 5.47)	0.338
	1–2 hours	191	158	**3.45 (1.42, 8.38)**	2.47 (0.81, 7.54)	0.112
	>2 hours	17	35	1.39 (0.49, 3.92)	1.21 (0.33, 4.47)	0.778
Perceived quality of care	Poor	265	156	**5.26 (3.63, 7.61)**	**3.66 (2.35, 5.69)**	**0.001**
	Good	52	161	1.00	1.00	
Knowledge of community-based health insurance	Low	107	43	**2.88 (1.82, 4.56)**	**6.02 (2.97, 12.26)**	**0.001**
	Medium	127	178	0.82 (0.57, 1.19)	1.698 (0.91, 3.18)	0.098
	High	83	96	1.00	1.00	
Active community participation	Yes	92	191	1.00	1.00	
	No	225	126	**3.71 (2.66, 5.16)**	**5.41 (3.29, 8.90)**	**0.001**
Chronic illness	Yes	57	88	1.00	1.00	
	No	260	229	**1.75 (1.20, 2.56)**	**10.82 (5.52, 21.21)**	**0.001**
Premium fee affordability	Yes	190	256	1.00	1.00	
	No	127	61	**2.81 (1.96, 4.01)**	**2.35 (1.47, 3.77)**	**0.001**
Non-reimbursed out-of-pocket money	Yes	78	38	**2.39 (1.57, 3.66)**	**9.37 (4.44, 19.77)**	**0.001**
	No	239	279	1.00	1.00	

*Shows a p-value less than 0.05; AOR, adjusted OR; COR, crude OR

Households having a low knowledge of CBHI is a significant risk factor for CBHI dropouts in age-categories of 20–29 years [(OR = 5.50 (95%CI = 1.21, 36.62)], 40–49 years [OR = 1.92 (95%CI: 1.08, 3.42), and 50–59 years [OR = 2.52 (95%CI: 2.00, 10.08) (Table 6); and the COR = 2.88 (95%CI: 1.82, 4.56) (Table 7). Nevertheless, households with low knowledge of CBHI have not shown any significant association with CBHI dropouts in 30–39 years. Hence, age is the other effect modifier for this study.

In contrast, those households with medium knowledge of CBHI are a significant protective factor from CBHI dropping outs in age categories of 20–29 and 40–49 years old. However, medium knowledge of CBHI has not shown any significant association with CBHI dropouts in the age categories of 30–39 and 50–59 years (Table 6). Hence, age is the other effect modifier for this study.

Those households who didn’t have active community participation is a significant risk factor for CBHI dropouts in age categories of 20–29 years [(OR = 4.59 (95%CI: 2.17, 9.75)], 30–39 years [OR = 4.77 (95%CI: 2.89, 7.85)], and 40–49 years [OR = 2.16 (95%CI: 1.19, 3.88)] (Table 6); and the COR = 3.71 (95%CI: 2.66, 5.16) (Table 7). Hence, age is the other effect modifier for this study.

Those households with no chronic illness were a significant factor for the dropouts from the CBHI scheme in the age categories of 30–39 years [(OR = 1.89 (95%CI: 1.04, 3.45), and 40–49 years [OR = 2.09 (95%CI: 1.14, 3.85) (Table 6); and the COR = 1.75 (95%CI: 1.20, 2.56) (Table 7).

Households who said the premium fee was not affordable was not a significant protective factor for the dropouts of the CBHI scheme in age categories of 20–29 years [OR = 0.34 (95%CI: 0.08, 1.37), 30–39 years [OR = 0.85 (95%CI: 0.38, 1.91), and 40–49 years [OR = 1.29 (95%CI: 0.59, 2.85) (Table 6); and COR = 2.81 (95%CI: 1.96, 4.01) (Table 7).

Similarly, those households who were not-reimbursed the spent out-of-pocket money is a significant risk factor for CBHI dropouts in the age categories of 20–29 years [(OR = 3.61 (95%CI: 1.29, 10.1), and 30–39 years [OR = 2.63 (95%CI: 1.34, 5.12) (Table 6); COR = 2.39 (95%CI: 1.57, 3.66) (Table 6). Hence, age is the effect modifier for this study.

*Income as a confounder/effect modifier*. Poor perceived quality of health care is a significant risk factor for CBHI dropouts in the first [OR = 2.68 (95%CI: 1.15, 6.24)] and fourth [OR = 2.05 (95%CI: 1.03, 4.10)] quartiles of income (Table 6); and the COR = 5.26 (95%CI: 3.63, 7.61) (Table 7).

Households with low knowledge of CBHI are a significant protective factor from CBHI dropping out in the third quartile [OR = 0.42 (95%CI = 0.19, 0.91)]; and the COR = 2.88 (95%CI: 1.82, 4.56) (Table 7). Similarly, households with medium knowledge of CBHI is a significant protective factor from CBHI dropping out in first [OR = 0.36 (95%CI = 0.14, 0.95)], second [OR = 0.86 (95%CI = 0.78, 0.94)], and third [OR = 0.43 (95%CI = 0.2, 0.91)] quartiles. However, households with medium knowledge are a significant risk factor for CBHI dropouts in the fourth quartile [OR = 3.71 (95%CI = 1.03, 13.1)] (Table 6). And the COR = 0.82 (95%CI: 0.57, 1.19) (Table 7).

Those households who did not have active community participation are a significant protective factor for CBHI dropouts in the third quartile [(OR = 0.02 (95%CI: 0.002, 0.125)] and fourth quartile [(OR = 82.08 (95%CI: 26.56, 253.67)] (Table 6); and the COR = 3.71 (95%CI: 2.66, 5.16) (Table 7). Hence, age is the other effect modifier for this study.

Those households who did not have a chronic illness were a significant protective factor for the dropouts from the CBHI scheme in the first quartile [(OR = 0.15 (95%CI: 0.07, 0.29), but is a risk factor in the third quartile (Table 6); and the COR = 1.75 (95%CI: 1.20, 2.56) (Table 7).

Households who said the premium fee was not affordable was not a significant protective factor for the dropouts of the CBHI scheme in the first [OR = 0.47 (95%CI: 0.23, 0.94), and third [OR = 0.33 (95%CI: 0.15, 0.71) quartiles; but it is a risk factor for forth quartile [OR = 11.35 (95%CI: 3.27, 39.36) (Table 6); the COR = 2.81 (95%CI: 1.96, 4.01) (Table 7).

Similarly, those households who were not-reimbursed the spent out-of-pocket money is a significant risk factor for CBHI dropouts in the first [(OR = 3.76 (95%CI: 1.83, 7.73) and second [OR = 21.3 (95%CI: 8.26, 54.78) quartiles; but it is a protective factor in the third [OR = 0.04 (95%CI: 0.005, 0.29) quartile (Table 6); COR = 2.39 (95%CI: 1.57, 3.66) (Table 7).

*Sex as a confounder/effect modifier*. Poor perceived quality of health care is a significant risk factor for CBHI dropouts in males [OR = 3.77 (95%CI: 2.57, 5.54)] (Table 6); and the COR = 5.26 (95%CI: 3.63, 7.61) (Table 7).

Households with low knowledge of CBHI is a significant risk factor for CBHI dropouts in males [OR = 3.38 (95%CI = 1.88, 6.09)]; but not significant in females [OR = 1.50 (95%CI = 0.57, 3.95)] (Table 6); the COR = 2.88 (95%CI: 1.82, 4.56) (Table 7). In contrast, households with medium knowledge of CBHI is a significant protective factor from CBHI dropping out in males [OR = 0.93 (95%CI = 0.90, 0.97)], and in females [OR = 0.45 (95%CI = 0.31, 0.65)] (Table 6); the COR = 0.82 (95%CI: 0.57, 1.19) (Table 7).

Those households who have no active community participation is a significant risk factor for CBHI dropouts in males [(OR = 4.49 (95%CI: 3.09, 6.51)] and in females [(OR = 3.02 (95%CI: 1.05, 8.64)] (Table 6); and the COR = 3.71 (95%CI: 2.66, 5.16) (Table 7).

Those households who have no chronic illness were a significant risk factor for CBHI dropouts in males [(OR = 1.58 (95%CI: 1.07, 2.34), but has no association with females (Table 6); and the COR = 1.75 (95%CI: 1.20, 2.56) (Table 7).

Households who said the premium fee was not affordable was not a significant risk factor of CBHI dropouts in males [OR = 2.12 (95%CI: 1.44, 3.11), and in females [OR = 17.5 (95%CI: 5.29, 57.87) (Table 6); the COR = 2.81 (95%CI: 1.96, 4.01) (Table 7).

Similarly, those households who were not-reimbursed the spent out-of-pocket money is a significant risk factor for CBHI dropouts in the males [(OR = 1.65 (95%CI: 1.04, 2.62) and females [OR = 20.11 (95%CI: 4.35, 92.99) (Table 6); COR = 2.39 (95%CI: 1.57, 3.66) (Table 7).

## Binary logistic regression analysis

In the univariate logistic regression analysis, Educational status, time spent to arrive at the nearby hospital, perceived quality of care, knowledge of community-based health insurance, active community participation, chronic illness, premium fee affordability, and non-reimbursed out-of-pocket money were the determinants for the dropout from community-based health insurance at a 20% level of significance. However, in the multivariable logistic regression analysis, perceived quality of care, knowledge of community-based health insurance, active community participation, chronic illness, premium fee affordability, and non-reimbursed out-of-pocket money were the determinants for the dropout from community-based health insurance at a p-value of < 0.05.

Accordingly, for those respondents who had a poor perceived quality of care, the odds of dropping out of CBHI were 3.7 (AOR = 3.66; 95%CI: 2.35, 5.69) times higher compared to those respondents who had a good perception of quality of care. Those respondents with low knowledge of community-based health insurance had a six-fold higher odds of dropping out of the CBHI scheme (AOR = 6.02; 95%CI: 2.97, 12.26) compared to those who had high knowledge of community-based health insurance.

Those respondents with no active community communication had a 5.4 (AOR = 5.41; 95%CI: 3.29, 8.90) higher chance of dropping out of CBHI than those with active community participation.

For those respondents who had no chronic illness, the odds of dropping out of CBHI were 10.8 (AOR = 10.82; 95%CI: 5.52, 21.21) times higher compared to those respondents who had a chronic illness.

Those who said the premium fee is not affordable had a 2.4 (AOR = 2.35; 95%CI: 1.47, 3.77) higher chance of dropping out of CBHI than those who said the premium fee is affordable.

For those respondents who had their out-of-pocket money not reimbursed, the odds of dropping out of the CBHI were 9.4 (AOR = 9.37; 95%CI: 4.44, 19.77) times higher than those respondents who had their out-of-pocket money reimbursed (Table 7).

## Discussion

The present study found that dropout from CBHI membership has been significantly associated with perceived quality of care, knowledge of CBHI, participation in scheme affairs, presence of chronic illness in the household, premium fee affordability, and non-reimbursement of out-of-pocket payments for health care services.

When household heads perceived the quality of care was poor, they were more likely to drop out of CBHI. This finding was supported by the study findings from the pilot districts of Ethiopia [17], Senegal [20], and Burkina Faso [23]; the quality of healthcare was identified as the most crucial determinant of dropout.

Similarly, the study participants who had poor knowledge of the CBHI program were more prone to drop out of the CBHI program. This finding was consistent with the studies conducted on various indigenous study findings in Ethiopia [2, 10, 17, 24], Senegal [20], Burkina Faso [23], and Tanzania [25]. There was the increasing importance of knowledge of insurance and a better understanding of the Scheme contributing to scheme retention [25].

Another factor contributing to the dropout was a lack of participation in CBHI program affairs, including holding a position. While participating in the CBHI program, there would be a chance to get detailed information on CBHI benefit packages and risk-sharing principles. But, on the contrary, those who didn’t participate would miss these opportunities, so they tend to drop out. The more active participation in the CBHI scheme, the stronger the statistically significant positive correlation with remaining enrolled [20].

Concerning the illness-related indicators, no chronic illness in the households had the most significant association with CBHI dropout. Hence, households with no experience of chronic illness were more likely to drop out than those with chronic illness. The reason might be that households with no chronic illnesses are not expected to visit healthcare facilities repeatedly for follow-ups and treatment options that may lead them to drop out of the CBHI scheme.

As a result, they may well not consider the unforeseeable illness in the future and instead opt out of the program. This notion was supported by research from Ethiopia [17], Sudan [26], Burkina Faso [23], and southwest India [27]. This condition could indicate that adverse selection was occurring, implying that only people in poor health were likely to renew their membership, whereas the healthiest individuals or households tended to leave the Scheme after enrolling, posing a threat to the long-term viability.

Another concern was the extent to which scheme costs drive dropouts. Disagreement on the affordability of premium fees was one of the factors driving dropouts. Since renewal is voluntary based on the Ethiopian community-based health insurance scheme context, when members perceive the premium fee is not affordable, they may cease to pay it and be forced to drop out. This finding was supported by a study conducted in Uganda, where the most significant obstacle was the inability to pay the premiums [23]. But studies conducted four years ago in pilot districts of Ethiopia [10, 17] and Senegal [20] found that premium payment did not have a statistically significant association with dropout. The difference might be because the premium fee was increased from 144 to 240 Ethiopian birr in the current study area.

Another factor influencing dropout was that none of the reimbursed out-of-pocket expenses were paid out of pocket. Individuals not reimbursed for out-of-pocket expenses were more likely to drop out of the CBHI program. Problems may arise when contracted health facilities, especially in hospitals, drug and laboratory reagents, stock out, so patients are often obliged to buy items from private retailers with no contact agreement with CBHI schemes.

Usually, services gained from private health facilities are more expensive than from government health facilities. Paying out of pocket is a problem for members who have already paid a registration fee and premium and may have no household budget for these contracted services. System-related challenges like the following are significant problems: Reimbursement requires the pharmacist to put a stamp on the back of the prescription and record/register it in the member patient’s file.

Usually, one of these procedures is not done because members are unaware of it. As a result, the member’s reimbursement claim is rejected, leaving the member highly disappointed and triggering their dropout. This evidence was supported by the evaluation of community-based health insurance pilot schemes in Ethiopia with key informant interviews; that states’ stock-outs in Pharmaceutical Fund and Supply Agency (PFSA) hubs have been the primary reason that health facilities give for their drug shortages [11]; and the study conducted in Tehuledere District [28].

### Strength of the study

Because this is a community-based study with large sample size, the results are highly inferable for the district. In the meanwhile, all of the cases were counselled to rejoin the community-based health insurance program. The study included both men and women, which could have shown the factors leading to CBHI scheme dropouts.

### Limitations of the study

Since the study was case-control, it is difficult to ascertain the identified determinants and CBHI scheme dropouts. There might also be a potential recall bias.

## Conclusions

The current study found that perceived low quality of health care, lack of knowledge about CBHI, lack of participation in CBHI scheme affairs, lack of chronic illness in the household, disagreement about premium fee affordability, and non-reimbursement of out-of-pocket payment for health care services were the determinants of household dropout from community-based health insurance in the study area.

Improving health care quality, educating the community about the CBHI program’s benefits packages and risk-sharing principles, reviewing and revising premium fee levels based on the ability to pay analysis and introducing different, stratified, and progressive premium levels rather than flat rates, designing an effective community involvement mechanism in decision making, and reimbursing members for out-of-pocket costs incurred due to referral to out-of-pocket providers.

## Supporting information

S1 File Part I: Socioeconomic and demographic characteristics. (DOCX)

S2 File (SAV)

## Acronyms/ abbreviations

AOR: adjusted odds ratio
CBHI: community-based health insurance
COR: crude odds ratio
OOP: Out of Pocket
PFSA: Pharmaceutical Fund and Supply Agency
SPSS: Statistical Package for Social Science
