# Supplementary material for: Determinants of household dropout from community-based health insurance program in northwest Ethiopia; A community-based case-control study
Source: PLoS One. 2023 Jan 11;18(1):e0276676. doi: 10.1371/journal.pone.0276676 (PMC9833576; doi:10.1371/journal.pone.0276676)
Supplement: S1 File — (DOCX) [file pone.0276676.s001.docx]

Part I: Socioeconomic and Demographic characteristics

| Q.code | Questions and filter | Coding categories | Skip |
| --- | --- | --- | --- |
| 001 | Renewal status | 1…renewed 2…non renewed |  |
| 001 | Sex of head of household | 1……male 2……female |  |
| 002 | What is your age | Age …………in years |  |
| 003 | What is your marital status | 1…….never married  2…….Married  3…….Widowed  4……..Divorced |  |
| 004 | What is your educational status | 1…….unable to read and write  2……. Able to read and write  3…….1-8 grade  4…….9-12 grade  5…….certificate and above |  |
| 005 | How many is your family size? | …… |  |
| 006 | What is your main occupation? | 1….. farmer  2….. merchant  3….daily laborer  4….. other |  |

**Part II -: Access to and perceived quality of care**

| QN | Questions and filter | Coding categories | | Skip |
| --- | --- | --- | --- | --- |
| 101 | How long you travel to reach nearest public health center? | 1= Less than 30 minutes  2=30 to 60 minutes  3= 1 to 2 hours  4=greater than 2hours | |  |
| 102 | How long you travel to reach nearest public hospital? | 1= Less than 30 minutes  2=30 to 60 minutes  3= 1 to 3 hours  4=3 to 6 hours  5= 6 hours and more | |  |
| 103 | How long did you wait to get consultation? | 1= Less than 30 minutes  2=30 to 60 minutes  3= 1 to 3 hours  4=3 to 6 hours  5= 6 hours and more | |  |
| 104 | How long did you wait to get diagnostic result? | 1= Less than 30 minutes  2=30 to 60 minutes  3= 1 to 3 hours  4=3 to 6 hours  5= 6 hours and more | |  |
|  | Part III: **perceived quality of care** | | | |
| 105 | Do health professionals treat CBHI members and non-CBHI Members equally? | | 1…….Yes  2……..No |  |
| 106 | Are health workers ethical? | | 1……yes  2…….no |  |
| 107 | Are health professionals competent? | | 1……yes  2…….no |  |
| 108 | Is there quality laboratory service? | | 1……yes  2…….no |  |
| 109 | Do all prescribed drugs available at the time of visiting health institutions? | | 1….yes  2…no |  |
| 110 | Is the health center far from your residence? | | 1….yes  2….no |  |
| 111 | Is getting service take long time? | | 1….yes  2…no |  |
| 112 | Did you get all the needed benefit packages at a time of visiting health institutions? | | 1…yes  2…no |  |
|  | **Part IV : knowledge and understanding of CBHI scheme** | | |  |
| 113 | Do you think you have enough information about the concept of CBHI? | | 1…yes  2…no |  |
| 114 | If yes, from whom you get the information about the insurance scheme? | | 1….From kebele mobilizes  2….health extension workers  3….from CBHI scheme workers 4…. Woreda cabins  5….Mass Media |  |
| 115 | Who can join in the program? | | 1. Formal sector employers  2. Informal sector working communities  3. Both  4. I don’t know |  |
| 116 | Who pays for insurance? | | 1. Household himself 2. Government 3. Third party 4. Other, specify |  |
| 117 | Who cover health service cost for members? | | 1. The scheme 2. Patient 3. Health service provider 4. Other, specify…………… |  |
| 118 | Only sick people buy CBHI? | | 1…yes  2…no |  |
| 119 | CBHI premium can be returned if your family did not get health service | | 1…yes  2…no |  |

**Part V: Active participation in the scheme**

| QN | Questions and filter | Coding categories | Skip |
| --- | --- | --- | --- |
| 201 | Have you ever participated in informal discussions about the scheme? | 1…yes  2….no |  |
| 202 | Have you ever participated in raising awareness /disseminating information about the scheme? | 1…yes  2…no |  |
| 203 | Have you ever participated in the scheme's general assembly meeting? | 1…….yes  2……..no |  |
| 204 | Have you ever participated in electing a leader of the scheme? | 1…….yes  2……..no |  |
| 205 | Have you ever received training under the scheme? | 1…….yes  2……..no |  |

**Part VI: Health status and health care utilization factors**

| QN | Questions and filter | Coding categories | Skip |
| --- | --- | --- | --- |
| 301 | Did any members of the household got illness/sick in the last twelve months? | 1……Yes  2……..No | If no skip Q 402 |
| 302 | Who got sick? | 1….Under five children  2…>65years old family members  3…..Head of households  4….other family members |  |
| 303 | Did any members of the household been ill with chronic illness which stays more than one year? | 1….yes  2….no | If no skip Q No.304 |
| 304 | If yes, specify the disease | 1. Hypertension 2. Diabetes mellitus 3. Asthma 4. cancer 5. Other, specify……… |  |
| 305 | Did any household member ever visit government health institutions? | 1….yes  2….no |  |
| 306 | Did you have ever getting your family members inpatient care? | 1….yes  2….no |  |

**Part-VII :scheme related factors**

| QN | Questions and filter | Coding categories | Skip |
| --- | --- | --- | --- |
| 401 | Do you think contribution payment rate for CBHI scheme is affordable? | 1…….yes  2……no |  |
| 402 | Do you have trust in management and administration of CBHI scheme? | 1…….yes  2……no |  |
| 403 | Do you think that renewal time is appropriate? | 1……yes  2……no | If no skip to Q No 306 |
| 404 | If your answer is **No** to Q 303, what is the reason? | 1..…It is not time that I get money  2….It burdened with other expenses  3… Other…… |  |
| 405 | Did you have not replaced OOP? | 1…yes  2…no | If no skip to Q No 306 |
| 406 | If yes, what was the reason given to you? | 1...unfulfilled documentation  2…did not follow referral procedure  3…deliance  4...other, state…… |  |
| 407 | Did you believe referral system is important? | 1…yes  2…no |  |
| 408 | If no, what was your reason? | 1..its complexity  2..health professionals delay to refer  3..it restricts us from using our preferred health institutions  4..other…. |  |

**Part VIII: Income status of households**

| S.N | Do you have these household assets? | Coding categories | How many of these household assets do this household currently own? | |
| --- | --- | --- | --- | --- |
| 501 | Plough oxen | 1….yes  2….no | ……. | |
| 502 | Cows | 1….yes  2…..no | …….. | |
| 503 | Goat | 1….yes  2…..no | ……. | |
| 504 | Sheep | 1….yes  2…..no | …….. | |
| 505 | Donkey | 1….yes  2…..no | ……. | |
| 506 | Horse | 1….yes  2…no | …….. | |
| 507 | Mule | 1…yes  2…no | ……. | |
| 508 | Chicken | 1….yes  2….no | ……. | |
| 509 | Beehives | 1….yes  2…no | ……. | |
| 510 | Crop Land( in hectare) | 1…..yes  2….no | ……. | |
| 511 | Crop land for irrigation | 1….yes  2….no | | ……Hectar |
| 512 | Land covered with eucalyptus(“bahir zaf”) | 1….yes  2….no | | …… |
| 513 | Maize in quintal per year | 1….yes  2….no | | ……………. |
| 514 | Teff in quintal per year | 1….yes  2….no | | …………… |
| 515 | Barley in quintal per year | 1….yes  2….no | | …………… |
| 516 | Wheat in quintal | 1….yes  2….no | | ……………….. |

**Thank you for your participation!**
